# A Case of Idiopathic Portal Hypertension Diagnosed by Noninvasive Fibrosis Evaluation Using Elastography

**DOI:** 10.7759/cureus.56432

**Published:** 2024-03-19

**Authors:** Rie Yano, Tomoko Tadokoro, Asahiro Morishita, Emi Ibuki, Tsutomu Masaki

**Affiliations:** 1 Gastroenterology and Neurology, Kagawa University, Kita-gun, JPN; 2 Diagnostic Pathology, Kagawa University, Kita-gun, JPN

**Keywords:** diagnosis, gastric varix, transient elastography, portal hypertension, idiopathic portal hypertension

## Abstract

Idiopathic portal hypertension (IPH) is often misdiagnosed as liver cirrhosis. Because it is difficult to distinguish between the two using diagnostic imaging, invasive tests, such as pathology and hepatic vein pressure gradient measurement, are necessary to make a diagnosis. Several studies have shown that the measurement of liver and spleen stiffnesses using elastography is useful in the diagnosis of IPH; however, there are few concrete reports on this subject. Herein, we report the case of a 58-year-old woman with IPH in which elastography was helpful for the diagnosis.

## Introduction

Idiopathic portal hypertension (IPH) is a disease characterized by splenomegaly, anemia, and portal hypertension without evidence of cirrhosis, extrahepatic portal vein or hepatic vein obstruction, or any other cause [1]. Its etiology is unknown and further elucidation of its pathology is required. Depending on its severity, IPH manifests as esophagogastric varices, portal hypertensive gastropathy, splenomegaly, anemia, and liver function disorders; however, it does not lead to liver cirrhosis (LC) or hepatocellular carcinoma. Transjugular or percutaneous liver biopsy examinations are essential for the diagnosis of IPH because IPH is indistinguishable from cirrhosis on radiological examination [2]. In addition to histopathological evaluation using liver biopsy, measurement of the hepatic venous pressure gradient (HVPG) using hepatic venography is necessary to differentiate IPH from LC. HVPG is the gold standard examination in the practice of portal hypertension, but it is highly invasive. HVPG is the pressure gradient between the portal vein and the inferior vena cava and is measured by a catheter inserted into the right jugular vein or other vein and passed through the right atrium. Both liver biopsy and HVPG measurement are invasive, and only a limited number of facilities can accurately diagnose them. IPH is often misdiagnosed as LC even in well-known centers for liver diseases. If IPH is diagnosed more conveniently using a diagnostic measure other than HVPG measurement or liver biopsy when cirrhosis is suspected, misdiagnosis can be reduced. Transient elastography transmits vibrations from the skin surface to the organs and quantifies the stiffness of the organs based on the speed of the transmission. The test can be repeated safely and painlessly, with only a light vibration being felt. Several studies have shown that measurement of liver and spleen stiffness using elastography is useful in the diagnosis of IPH, but there are few specific case reports. Herein, we describe a case of IPH in which liver and spleen elastography was useful for differentiating IPH from LC.

## Case presentation

A 58-year-old woman was referred to our hospital for a close examination of pancytopenia and gastric varices. She had been diagnosed with pancytopenia 30 years previously. Since then, annual physical examinations have indicated pancytopenia; however, there has been no history of close examinations. When she visited a family physician because of a positive fecal occult blood test on medical checkup, pancytopenia, gastric varices, and splenomegaly were found, and she was referred to our hospital. On admission, she had no history of blood transfusion or drug allergy and did not consume alcohol. There was a family history of liver disease with unknown details in her mother and rheumatoid arthritis in her sister. Her physical status and vital signs on admission were as follows: height, 149 cm; bodyweight, 39.4 kg; body mass index, 17.7 kg/m^2^; blood pressure, 125/82 mmHg; and pulse, 59 bpm (regular). The patient was fully conscious, and no remarkable changes were observed in the heart or lungs. Her abdomen was flat with no tenderness, and bowel peristalsis was evident. No edema was observed in the extremities. The laboratory investigations showed the following results (Table 1).

**Table 1:** Laboratory data of patients WBC - white blood cell, RBC - red blood cell, Hb - hemoglobin, Ht - hematocrit, Plt - platelet, Neut - neutrophil, Eos - eosinophil, Baso - basophils, Lym - lymphocyte, Mono - monocyte, PT - prothrombin time, PT-INR - prothrombin time-international normalized ratio, APTT - activated partial thromboplastin time, TP - total protein, Alb - albumin, T.Bil - total bilirubin, AST - aspartate aminotransferase, ALT - alanine aminotransferase, ALP - alkaline phosphatase, γ-GTP - γ-glutamyl transpeptidase, LDH - lactate dehydrogenase, BUN - blood urea nitrogen, Cr - creatinine, HBs - hepatitis B surface, HBc - hepatitis B core, HCV - hepatitis C virus, AFP - alfa-fetoprotein, DCP - des-g-carboxy prothrombin, CEA - carcinoembryonic antigen, CA19-9 - carbohydrate antigen 19-9, M2BiGi - Mac-2 binding protein glycosylation isomer, HbA1c - glycated hemoglobin, Ig - immunoglobulin, ANA - antinuclear antibody, AMA - antinuclear antibody, Fe - iron, TIBC - total iron binding capacity, UIBC - unsaturated iron binding capacity

Metric	Value	Unit	Reference
WBC	1970	/µl	4700-8700
RBC	433x10^4^	/µl	370-490
Hb	11.2	g/dl	11-15
Ht	36.2	%	35-45
Plt	7.4x10^4^	/µl	15-35
Neut	64.5	%	38-71.9
Eos	2.0	%	0.2-6.8
Baso	0.0	%	0-1
Lym	28.5	%	26-46.6
Mono	5.5	%	2.3-7.7
PT	94	%	80-100
PT-INR	1.01		0.85-1.15
APTT	43.6	second	24.0-32.0
D-Dimer	0.7	µg/mL	0.0-1.0
TP	7.3	g/dl	6.5-8.2
Alb	4.1	g/dl	3.5-5.5
T-Bil	0.89	mg/dl	0.1-1.2
D-Bil	0.7	mg/dl	0.1-0.6
AST	29	U/l	10-35
ALT	16	U/l	5-40
ALP	71	U/l	100-340
γ-GTP	25	U/l	0-30
LDH	161	U/l	110-220
BUN	9.0	mg/dl	8.0-20.0
Cr	0.52	mg/dl	0.46-0.79
HBs Antigen	negative	IU/ml	0-0.05
HBs Antibody	negative	mIU/mL	-1
HCV Antibody	negative	S/CO	0-1
AFP	2	ng/ml	0-9
DCP	12	mAU/ml	0-40
CEA	3.1	ng/mL	0.0-5.0
CA19-9	2	ng/mL	0-37
M2BPGi	2+		-1
C.O.I	3.00		0-1
Autotaxin	1.429	mg/L	0-1.270
Type IV collagen 7S	5.2	ng/mL	0-4
Hyaluronic acid	63.2	ng/ml	0-50
HbA1c	4.9	%	4.9-6.0
IgA	451	mg/dL	93-393
IgG	1579	mg/dL	861-1747
IgM	251	mg/dL	50-269
ANA	640		0-40
Homogeneous			
Speckled			
AMA	20		0-20
AMA-M2	negative		-1
M2 index	1.5		0.0-7.0
Fe	56	µg/mL	40-188
TIBC	321	µg/mL	
UIBC	265	µg/mL	137-325
Ferritin	57.7	ng/mL	6.0-138.0

**Figure 1:**
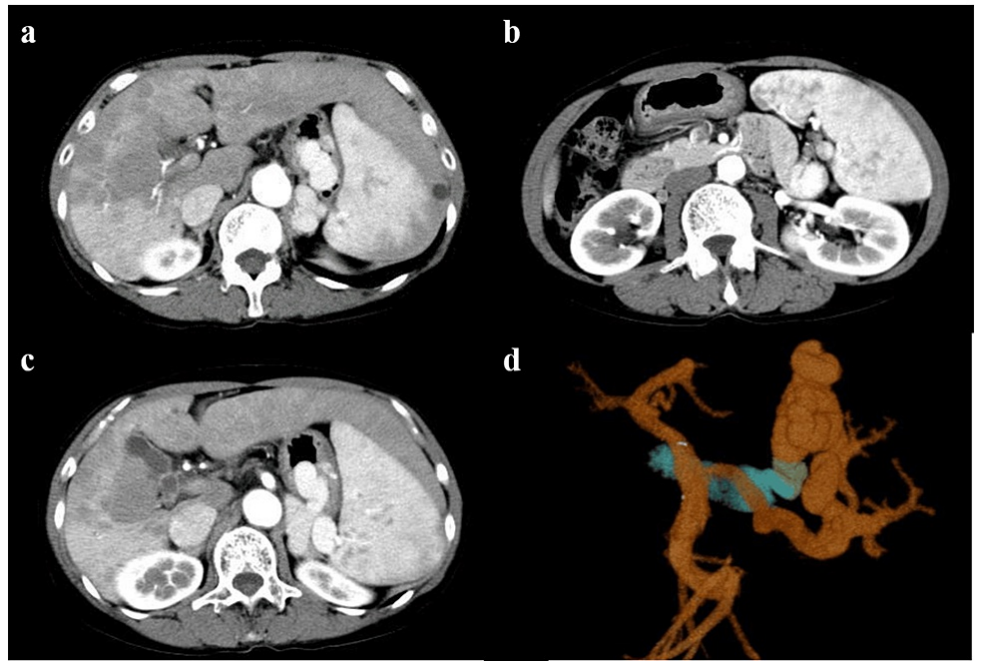
Abdominal contrast-enhanced computed tomography (CT) scan in the arterial phase a. Heterogeneous contrast effect is noted in the liver, the intrahepatic portal vein is narrowed, and a small mural thrombus is observed in some areas; b. There is marked splenomegaly and the splenic veins are dilated; c, d. Gastric varices are seen in the gastric vault. The short gastric vein is the main blood supply draining into the gastrorenal shunt.

Genetic testing results were negative for calreticulin (CALR) Type 1/2, Janus activating kinase 2 (JAK2) V617F, and myeloproliferative leukemia (MPL) W515L/K. Therefore, myeloproliferative diseases were unlikely. Autoantibodies were positive for ANA, anti-SS-A antibodies, and anti-Scl-70 antibodies; however, examination by a collagen specialist did not lead to a definitive diagnosis of scleroderma or other collagen diseases because the patient had no skin or joint symptoms. Abdominal ultrasonography revealed blunted margins of the liver, markedly coarsened parenchyma, and splenomegaly (spleen index, 81.54 cm^2^). Abdominal contrast-enhanced CT revealed heterogeneous contrast effects in the liver parenchyma, splenomegaly, collateral vessels, gastric varices, and dilation of the splenic artery. The gastric varices were supplied by the short gastric vein and drained into the gastrorenal shunt. There was no obstruction in the extrahepatic portal vein, including the porta hepatis (Figure 1).

**Figure 2:**
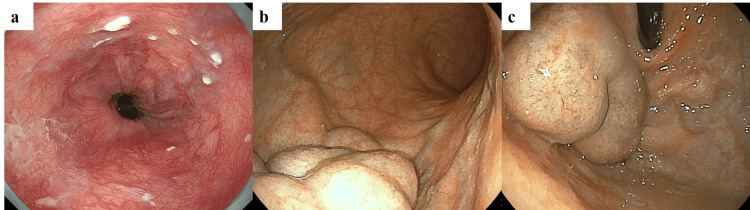
Upper gastrointestinal endoscopy a. Straight and thin esophageal varices are observed in the lower esophagus, which are blue in color, with no signs of red color or bleeding; b, c. Nodular or mass-like gastric varices present continuously from the gastric vault to the body. These are white in color, with no signs of red color or bleeding.

Based on the general rules for recording the endoscopic findings of esophagogastric varices in Japan [3], upper gastrointestinal endoscopy showed F1 type esophageal (LiF1CbRC0) and F3 type gastric (Lg-bF3CwRC0) varices (Figure 2).

**Figure 3:**
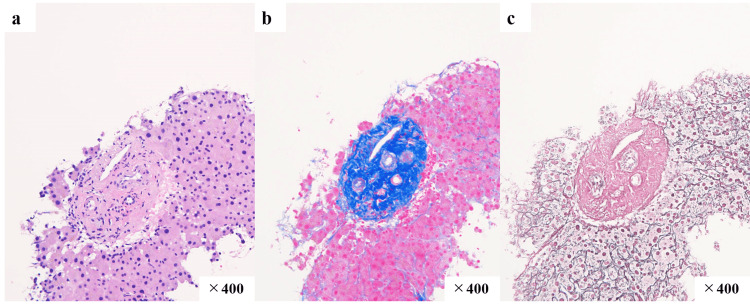
Histopathological findings The portal area was fibrotic and enlarged, with a scattered number of similarly rounded areas a. Hematoxylin-eosin staining; b. Azan staining; c. Silver impregnation

Abdominal ultrasonography and CT scan were inconclusive of IPH. Liver stiffness was measured by transient elastography (FibroScan®, Integral Corporation, Tokyo, Japan), and a controlled attenuation parameter (CAP) of 171 dB/m (equivalent to S0) and liver stiffness measurement (LSM) of 6.6 kPa (equivalent to F0-1) by vibration-controlled transient elastography (VCTE). On the other hand, splenic stiffness was measured by transient elastography, and VCTE was 72.4 kPa. The results of transient elastography indicated that the patient was unlikely to have cirrhosis and suggested another cause of portal hypertension. Percutaneous liver biopsy was performed for histopathological diagnosis. The liver biopsy specimen showed that the portal region was circularly fibrotic and enlarged. No portal vein obstruction or thrombus formation was observed. The fatty deposits were less than 5%. There was no bile duct loss. There was little evidence of fibrosis or inflammation in the liver parenchyma (Figure 3).

**Figure 4:**
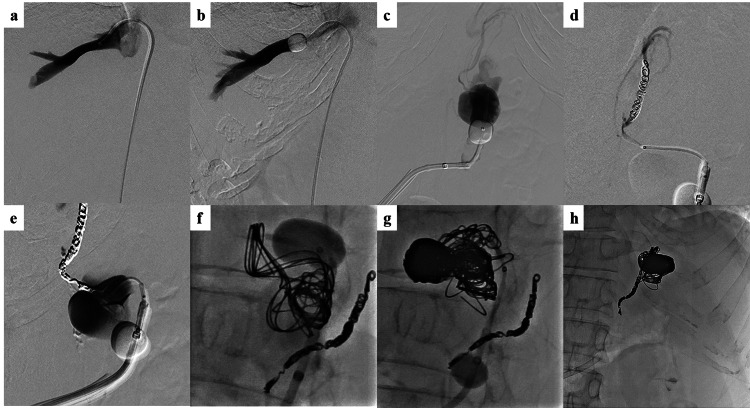
Angiography findings a, b. A catheter is placed in the right hepatic vein and free hepatic vein pressure and occluded hepatic vein pressure are measured. There is no obstruction or stenosis of the hepatic veins; c. Gastrorenal shunt and drainage pathway to the left inferior transverse vein; d, e, f, g, h. Coil embolization is performed in the left inferior transverse vein and varicose veins

A liver biopsy ruled out cirrhosis, autoimmune hepatitis, and primary biliary cirrhosis. The hepatic venous pressure gradient (HVPG) was measured (Figure 4). On hepatic venography, the wedge hepatic vein pressure (WHVP) was 5 mmHg, free hepatic vein pressure (FHVP) was 0 mmHg, and WHVP-FHVP was calculated to be 5 mmHg (reference, 1-5 mmHg). Normal HVPG ruled out portal hypertension due to sinusoidal portal hypertension [4].

**Figure 5:**
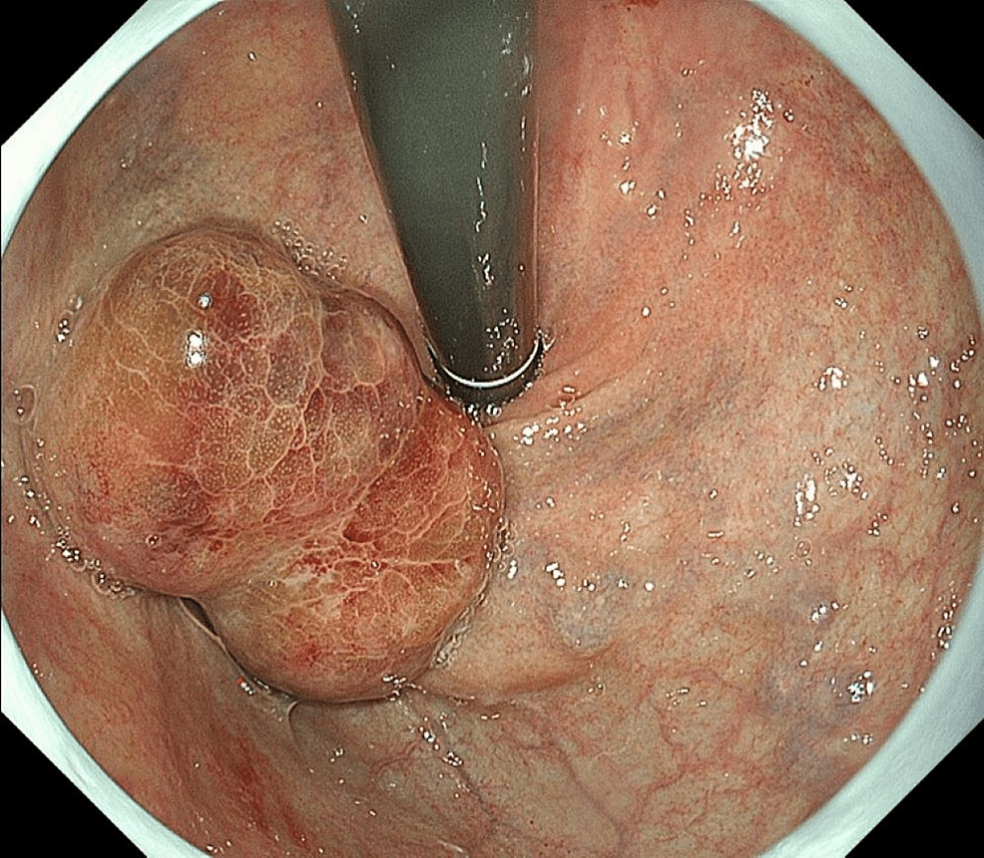
Gastrointestinal endoscopy after BRTO After BRTO, upper gastrointestinal endoscopy shows that the gastric varices are discolored and reduced in size. BRTO, balloon-occluded retrograde transvenous obliteration

Based on these test results, the patient was diagnosed with IPH. The patient underwent balloon-occluded retrograde transvenous obliteration (BRTO) of gastric varices. After BRTO, upper gastrointestinal endoscopy revealed that the gastric varices were smaller and discolored, indicating a good therapeutic effect (Figure 5).

## Discussion

IPH is characterized by portal hypertension with overt splenomegaly, pancytopenia, and relatively mild abnormalities of liver function and morphology. IPH is a rare cause of portal hypertension and is frequently misdiagnosed as cirrhosis [1]. However, the etiology of IPH is not fully understood. IPH is a disease that is characterized by splenomegaly and portal hypertension. Occlusion of the peripheral portal veins in the liver and increased splenic blood flow due to splenomegaly have been reported to cause portal hypertension. However, as some cases are complicated by autoimmune diseases, some reports have pointed to immunological involvement [5].

IPH is diagnosed comprehensively based on general laboratory findings, such as blood tests, imaging findings, such as ultrasonography, liver stiffness measurement (LSM), angiography, and pathological findings. Additionally, other diseases that cause portal hypertension, such as cirrhosis, extrahepatic portal vein obstruction, and Budd-Chiari syndrome, should be ruled out. The HVPG is elevated in portal hypertension due to cirrhosis. HVPG is the pressure gradient between the portal vein and inferior vena cava; normal HVPG is 1-5 mmHg. In cirrhosis, the clinically important threshold for the HVPG is 10 mmHg, above which there is progression of ascites and esophageal varices; above 12 mmHg, the risk of variceal rupture increases [6]. Conversely, it has been reported that in HVPG <10 mmHg, the liver remains in compensated cirrhosis for four years or more [7]. In contrast, the HVPG is only normal to mildly elevated (<10 mmHg) in IPH [8]. In a previous report, the mean HVPG was 7.1 ± 3.1 mmHg in patients with IPH and was markedly lower than that in those with cirrhosis (17 ± 3 mmHg) [9]. Although HVPG measurement is very useful in differentiating between IPH and cirrhosis, the procedure involves invasive catheterization and is impractical to repeat. Moreover, the number of facilities that can accurately perform the HVPG measurement is limited. A liver biopsy is also necessary to diagnose IPH. Histopathological changes in the portal tract commonly include capillary dilatation, phlebosclerosis, and fibroelastosis of the stroma [10]. In this case, the liver biopsy specimen showed well-defined, round fibrosis in the portal region, which is a characteristic of IPH. A detailed evaluation of the abnormalities, including the presence of abnormal blood channels, was difficult because of tissue fragmentation; however, the findings were consistent with IPH.

Ultrasound elastography was first developed in 1992 with transient elastography as a method to measure clinical liver fibrosis [11]. It uses the propagation of shear waves at 50 Hz using a mechanical impulse. The median (kPa), success rate (%), and interquartile range (IQR)/median of the elasticity values measured 10 times are displayed and can be considered reliable if the success rate is >60% and the IQR /median is <0.3 (30%) [12]. Simultaneously, the CAP can measure fat attenuation. Shear wave (SWE) involves excitation by an acoustic radiation force impulse (ARFI). When ultrasound with a long pulse length (push pulse) irradiates a living body, the tissue is pushed backward by the ARFI and then returns to its original position, generating transverse waves (shear waves). The propagation velocity of shear waves in the tissue can be measured. Transient elastography and other elastographic procedures are recommended to diagnose the stages of fibrosis in cirrhosis, and the number of liver biopsies is decreasing [13]. Reports on the diagnosis of portal hypertension are also increasing [14]. In recent years, elastography has become widely used as a noninvasive method for evaluating liver fibrosis and portal hypertension, and its usefulness has been further demonstrated in differentiating between IPH and LC, as in this case.

Seijo et al. reported a lower mean liver stiffness in IPH by transient elastography (8.4 ± 3.3 kPa) than in cirrhosis (40.9 ± 20.5 kPa) [9]. Liver stiffness, in this case, at the first visit was 6.6 kPa, which is much lower than that in patients with LC. Furuichi et al. reported that measuring the spleen/liver stiffness ratio using SWE made it possible to diagnose IPH non-invasively, specifically, and accurately [15]. Even in the measurement of SWE, splenic stiffness is considered to have higher results than hepatic stiffness [15]. The stiffnesses of the liver and spleen on transient elastography at the initial examination of our patient were 6.6 kPa and 72.4 kPa, respectively. There is no reference value for the spleen/liver stiffness ratio by FibroScan® yet, but it appeared to be elevated in this case, and the results are considered consistent with IPH. Recently, a novel spleen-dedicated FibroScan® at 100 Hz has been reported to be useful for measuring spleen stiffness [16]. The FibroScan® used in this case was of 50 Hz, and we are considering using a dedicated machine in the future. The Virtual Baveno VII Consensus Workshop recommends upper gastrointestinal endoscopy for varices screening in patients with compensated cirrhosis who have liver stiffness >20 kPa or platelet count <150,000/µL and are not taking nonselective beta-blockers. Conversely, patients with liver stiffness <20 kPa and platelet counts >150,000/µL are unlikely to have varices requiring treatment and may choose not to undergo endoscopic screening [17]. The liver stiffness at the time of the initial examination of our patient was 6.6 kPa (<20 kPa) but the platelet count was low at 74,000/μL, a result that recommended upper gastrointestinal endoscopy. Although liver stiffness is often elevated in patients with cirrhosis in general, and upper gastrointestinal endoscopy is often performed when liver stiffness exceeds 20 kPa, it should be noted that even patients who do not have elevated liver stiffness, as in this case, may have massive gastric varices.

In this case, although LC was suspected based on various fibrosis markers and imaging studies, IPH was suggested based on the normal liver stiffness measured using ultrasound elastography. Ultimately, IPH was diagnosed using liver biopsy and HVPG measurements. Although the prognosis of IPH is better than that of LC, rupture of gastroesophageal varices and pancytopenia due to splenomegaly are often problematic. Therefore, IPH treatment consists of treatment and follow-up of such comorbidities. As this patient had gastric varices with a high risk of rupture, treatment of these varices was the primary priority. Splenectomy will be considered if uncontrolled pancytopenia or portal hypertension develops in the future. In addition, the incidence of hepatocellular carcinoma is less common than that of LC, but follow-up is important.

## Conclusions

This is an important case in which ultrasound elastography allowed us to suspect IPH. Clinicians should consider ultrasound elastography for the initial evaluation of suspected portal hypertension. It is less invasive, reproducible, and useful for the evaluation of liver fibrosis and the diagnosis and follow-up of IPH.
